# Incidence and Risk Factors of Thyroid Malignancy in Patients with Toxic Nodular Goiter

**DOI:** 10.1155/2022/1054297

**Published:** 2022-05-23

**Authors:** Tarek Zaghloul Mohamed, Ahmed Abd El Aal Sultan, Mohamed Tag El-Din, Ahmed A. Elfattah Mostafa, Mohammed A. Nafea, Abd-Elfattah Kalmoush, Mohammed Shaaban Nassar, Mohamad Adel Abdalgaleel, Ahmed M. Hegab, Ayman Helmy Ibrahim, Mohamad Baheeg

**Affiliations:** ^1^General Surgery, Faculty of Medicine, Al-Azhar University, Cairo, Egypt; ^2^Surgical Oncology, Faculty of Medicine, Al-Azhar University, Cairo, Egypt

## Abstract

**Background:**

Although hyperfunctioning thyroid disorders were thought to be protective against malignancy, some recent studies reported a high incidence of incidentally discovered cancer in patients with hyperfunctioning benign thyroid disorders. We performed this study to estimate the incidence and predictors of malignant thyroid disease in patients with toxic nodular goiter (TNG). *Patients and Methods*. The data of 98 patients diagnosed with TNG were reviewed (including toxic multinodular goiter SMNG and single toxic nodule STN). The collected data included patients age, gender, systemic comorbidities, family history of thyroid malignancy, previous neck radiation, type of disease (multinodular or single), size of the dominant nodule by the US, operative time, and detection of significant lymph nodes during operation. Based on the histopathological analysis, the cases were allocated into benign and malignant groups.

**Results:**

Malignancy was detected in 21 patients (21.43%). Although age distribution was comparable between the two groups, males showed a significant increase in association with malignancy. Medical comorbidities and family history of cancer did not differ between the two groups. However, TMNG showed a statistically higher prevalence in the malignant group. Operative data, including operative time and lymph node detection, were comparable between the two groups. On regression analysis, both male gender and TMNG were significant predictors of malignancy.

**Conclusion:**

The presence of thyroid hyperfunction is not a protective factor against malignancy, as malignancy was detected in about 1/5 of cases. Male gender and TMNG were significant risk factors of malignancy in such patients.

## 1. Introduction

The incidence of thyroid dysfunction is increasing around the world, as approximately they represent 30–40% of patients visiting endocrine clinics, making it one of the most common endocrine disorders [1, 2]. According to a previous Egyptian epidemiological study, about 30% of patients attending the endocrine clinic had thyroid dysfunction, from whom 19.2% had hyperthyroidism, and 15.8% had subclinical hyperthyroidism [2].

Graves' disease, toxic multinodular goiter (TMNG), and single toxic nodule (STN) are the most common etiologies of hyperthyroidism [3, 4].

The increased levels of thyroid-stimulating hormone (TSH) were reported to increase the risk of thyroid malignancy, even within normal ranges. Additionally, it is associated with more advanced nodular thyroid disease [5]. TSH stimulates the proliferation of both normal and well-differentiated neoplastic thyroid tissues. Based on the previous belief, suppression of this hormone is one of the management plans for patients with well-differentiated thyroid malignancy [6].

Patients with primary toxic goiter express decreased levels of TSH. As a result, they are less likely to harbour malignant disease due to inhibition of the related oncogenes [7]. Based on these data, the presence of hyperfunctioning thyroid disorder was thought to be protective against malignancy [4].

Older studies reported a very low incidence of thyroid cancer in patients with hyperfunctioning thyroid nodules (3–5%) [8]. Even the American Thyroid Association does not recommend cytological evaluation for such nodules due to low malignancy risk [3]. Nevertheless, recent evidence has proved increased incidence of thyroid malignancy in toxic goiter patients (12–18%), which confirms that this incidence has been underestimated in the past [4, 9].

Herein, we performed this study to estimate the incidence and predictors of malignant thyroid disease in patients with toxic nodular goiter (TNG).

## 2. Patients and Methods

This is a retrospective study that was performed at the General Surgery Department, Al-Azhar University Hospitals. We retrospectively reviewed the data of consecutive 98 adult patients diagnosed with TNG who underwent surgical intervention during the period between January 2018 and December 2020. TNG was defined when the patient had thyroid nodule (single or multiple), decreased serum TSH, with normal or increased T3 or T4 [4].

All of the included cases were subjected to history taking, clinical examination, and routine preoperative laboratory investigations (including TSH, T3, and T4). Additionally, neck ultrasonography (US) was ordered for all cases, and the detected nodules were classified according to the British Thyroid Association (BTA) classification [10]. We included only patients who were classified as U2 according to the previous classification and did not require preoperative fine-needle aspiration cytology (FNAC). Contrarily, patients with U3 (intermediate), U4 (suspicious), or U5 (malignant) were excluded from the study. Also, we excluded patients with Graves' disease or previous history of neck radiation. Only cases diagnosed with single nodule were subjected to thyroid scan to confirm hyperfunction.

After explaining the benefits, details, and potential problems of surgery, we obtained signed informed permission from all patients prior to surgical intervention. All cases underwent total thyroidectomy following normalization of their thyroid profile, according to our management protocol. The surgical specimen was sent to the histopathology laboratory for analysis. Incidental thyroid cancer was established when discovered on histopathological analysis in patients lacking the clinical features of malignancy [11]. In our department, routine thyroid evaluation included analysis of all dominant nodules and obtaining one representative section from every one cm of the remaining thyroid tissue. Based on the final histopathological report, we classified the included patients into two groups; benign and malignant.

The collected data included the patient's age, gender, systemic comorbidities, family history of thyroid malignancy, previous neck radiation, type of disease (multinodular or single), size of the dominant nodule by the US, operative time, and detection of significant lymph nodes during operation.

### 2.1. Statistical Analysis

The Statistical Package for the Social Sciences (SPSS 26, IBM/SPSS Inc., Chicago, IL) software was used to analyse the collected data. Frequencies and percentages (%) or mean values with standard deviations (SD) were used to report basic demographic statistics (SD). The chi-square test was used to compare two independent sets of qualitative data. To compare quantitative data, the independent-samples *t*-test and the Mann–Whitney *U* test were utilised. Risk variables for the binary categorical outcome were examined using univariate and multivariate logistic regression. A *p* value of 0.05 or less was used to determine the statistical significance.

## 3. Results

The mean age of the cases included in the study was 47.13 years in the benign group and 48.67 years in the malignant group, with no significant difference between the two groups. Although the female gender was predominant in the two groups, males showed a significant increase in association with malignancy (*p*=0.023).

The prevalence of systemic comorbidities, including diabetes, hypertension, and chronic liver disease, showed no significant difference between the study groups. Likewise, positive family history of thyroid cancer was reported only in one case (1.3%) in the benign group, and that was statistically comparable to the malignant one.

We included two types of TNG: TMNG and STN. The former had a statistically larger prevalence in the malignant group, as it was found in 57.14 percent of cases in the benign and 80.95 percent of cases in the malignant group (*p*=0.046). On the other hand, the size of the dominant nodule, measured by the US, did not show significant differences between the two groups (*p*=0.303). The previous data are shown in Table 1.

When it comes to the operative data, operative time was statistically comparable between the two groups (87.17 vs 93.29 in the benign and malignant groups;*p*=0.204). No significant lymph nodes were detected on surgical exploration in neither of the two groups.  Table 2 illustrates these data.

On regression analysis to detect risk factors of malignancy in TNG patients, both male gender and TMNG were significant predictors of malignancy in patients with TNG in univariate and multivariate analyses, as shown in Table 3.

### 3.1. CI: Confidence Interval

Regarding the detected malignancies in our study, 9 patients were diagnosed with papillary cancer (42.86%) and 7 patients had the follicular variant of papillary cancer (33.33%), while the remaining five cases had the follicular type (23.81%) (data not shown in tables).

## 4. Discussion

Historically, the hyperthyroid state observed in patients with TNG was presumed to be “protective” against malignant thyroid neoplasms [12]. However, there is a current debate in the existing literature [6], as some studies reported very low incidence [8], while others reported a high incidence of malignancy in TNG patients [4]. According to a recent meta-analysis published in 2021, hot nodules had a lower risk of cancer than cold nodules. Nonetheless, the rate of malignancy in hot nodules was higher than anticipated [13].

This study was conducted to estimate the incidence and risk factors of malignant thyroid disease in patients with TNG. We included a total of 98 patients diagnosed with TNG. After histopathological examination of the surgical specimen, 21 patients appeared to harbour thyroid malignancy, with an incidence rate of 21.43%.

This is consistent with a number of recent studies that have found an incidence rate similar to ours. Smith et al. reported that the rate of thyroid malignancy in their 164 patients was 18.3% (30 patients) [4]. Moreover, Tam et al. reported an incidence rate of 19.2%, as 14 patients had malignancy out of the included 73 cases [6].

Other studies reported a lower incidence of the same parameter. Giles et al. reported an incidence rate of 12%, whereas Cerci et al. reported that malignancy was detected in 11 out of 124 patients with TMNG (incidence = 9%) [14]. Kang et al. included cases with TMNG and STN like us, and they detected malignancy in five out of the included 181 cases, with an incidence rate of 2.7% [8].

A previous study also emphasized that hyperfunctioning nodules in the pediatric population have a higher malignancy risk, reaching up to 29% [15]. This was not observed in our study as all of our cases were adults (>18 years).

Apparently, there is some heterogenicity regarding the incidence between different studies, and this could be explained by different patient selection criteria, aetiology of hyperthyroidism, the operation performed (total or hemithyroidectomy), or the extent of histopathological examination [16, 17].

In the current study, patient age was not a significant risk factor for malignancy, as it had mean values of 47.13 and 48.67 years in the benign and malignant groups, respectively, with no significant difference in statistical analysis. Tam et al. confirmed our findings regarding age, as it was statistically comparable between benign and malignant groups (*p*=0.416). The included patients had median values of 49 and 50 years in the benign and malignant groups, respectively [6]. Other authors confirmed the previous findings regarding age (*p*=0.382) [18].

In our study, the male gender was a significant predictor of malignancy on both univariate and multivariate analyses. Males represented 15.58% and 38.1% of the included cases in the benign and malignant groups, respectively. In line with our findings, another study reported that the male gender could be a risk factor for malignancy in such cases. Males represented 27% of patients in the malignant group, compared to only 11% in the benign group (*p*=0.03) [4]. Contrarily, another study negated any significant impact of gender on the incidence of cancer, as males represented 44% and 50% of patients in the benign and malignant groups, respectively (*p*=0.77) [6].

Our findings showed that positive family history of thyroid cancer did not have a significant impact on harbouring malignancy in patients with TNG. Another study also confirmed the previous findings regarding positive family history of thyroid cancer [4].

In the current study, TMNG was a significant risk factor for thyroid malignancy on univariate and multivariate analyses. It was present in 57.14% and 80.95% of patients in the benign and malignant groups, respectively. Another study confirmed our findings regarding TMNG, as the incidence of malignancy in patients with TMNG was 21%, compared to only 4.5% in patients with STN (*p*=0.04) [4]. Some authors have also reported an increased risk of cancer in patients with thyroiditis [19], but we did not include such patients in our study.

In the current study, the size of the dominant thyroid nodule was not a significant risk factor of malignancy, as it had comparable mean values between the benign and malignant groups (*p*=0.303). Likewise, Tam et al. reported comparable nodule diameters between the benign and malignant groups (*p*=0.413). It had median values of 41.5 and 35 mm in the benign and malignant groups, respectively [6].

In our study, operative parameters including duration of surgery and detection of significant lymph nodes showed no significant difference between the two groups. This is probably that these cases had early-stage cancers that were not associated with lymph node metastasis or any other operative difficulty that might prolong operative time.

When analyzing malignant specimens detected in the current study, 9 patients were diagnosed with papillary cancer (42.86%) and 7 patients had the follicular variant of papillary cancer (33.33%), while the remaining five cases had the follicular type (23.81%). Smith et al. also reported that most of the accidently discovered cases had papillary cancer (73%), while the remaining cases had the follicular variant of papillary carcinoma [4]. In the study conducted by Mirfakhraee et al., the authors reported that papillary neoplasm was the most common malignancy detected in patients with hyperfunctioning thyroid nodules as it was present in 57.1% of malignant cases [20]. On the other hand, Als et al. reported that follicular neoplasm was the most common type, as it was detected in 15 out of 19 patients with TNG (79%) [21]. This contradicts the previous findings.

Whether being papillary or follicular, the high incidence of malignancy in toxic thyroid patients should alert the surgical community towards changing our management plan for such cases. Patients who have SNG with documented risk factors of malignancy should be scheduled for surgery to catch malignancy at early stages, and we suggest that patients should potentially need preoperative FNA for dominate nodules to rule-out malignancy, which will change the timing of surgery to catch malignancy at early stages, and protect patients with papillary thyroid carcinoma from neck LNs dissection, as reported by Hisham Omran et.al. that total thyroidectomy without prophylactic central LN dissection in early papillary thyroid cancer with no statistical difference as regard to postoperative recurrence especially with presence of postoperative radioactive ablation [22].

Our study has some limitations such as being retrospective in nature, conducted in a single-centre, and the relatively small sample size. Hence, more studies including more patients from different surgical centres should be conducted to establish a risk stratification system for these patients.

## 5. Conclusion

According to the previous results, the presence of thyroid hyperfunction is not a protective factor against malignancy, as malignancy was detected in about 1/5 of these cases. Male gender and TMNG were significant risk factors of malignancy in such patients. Patients who have SNG with documented risk factors of malignancy should be scheduled early for surgery to catch malignancy at early stages, which will affect the need for further surgery in the setting of thyroid malignancy. So, we suggest that patients should potentially need preoperative FNA for dominate nodules even in the presence toxicity to rule-out malignancy, which will change the timing of surgery to catch malignancy at early stages.

## Figures and Tables

**Table 1:** Preoperative data.

Variable	Benign group (*n* = 77)	Malignant group (*n* = 21)	*p* value
Age (year)	47.13 ± 11.94	48.67 ± 10.66	0.594
Gender			
Male	12 (15.58%)	8 (38.1%)	0.023^*∗*^
Female	65 (84.42%)	13 (61.90%)	
Comorbidities			
Diabetes mellitus	9 (11.69%)	3 (14.29%)	0.748
Hypertension	9 (11.69%)	4 (19.05%)	0.378
Chronic liver disease	2 (2.6%)	0 (0%)	0.456
Family history of cancer thyroid	1 (1.3%)	0 (0%)	0.600
Preoperative diagnosis			
TMNG	44 (57.14%)	17 (80.95%)	0.046^*∗*^
STN	33 (42.86%)	4 (19.05%)	
Size of the dominant nodule (mm) by the US	32.29 ± 12.93	35.67 ± 14.43	0.303

**Table 2:** Operative findings.

Variable	Benign group (*n* = 77)	Malignant group (*n* = 21)	*p* value
Operative time	87.17 ± 19.30	93.29 ± 19.90	0.204
Detected lymph nodes	0 (0%)	0 (0%)	1

**Table 3:** Regression analysis to detect risk factors of malignancy in TNG.

Variables	Univariate analysis	Multivariate analysis		
		OR	95% CI for OR	*p* value
Age	0.590			
Male gender	0.028^*∗*^	2.713	1.207–8.423	0.043
Diabetes	0.748			
Hypertension	0.383			
Chronic liver disease	0.999			
Family history of cancer thyroid	0.999			
TMNG	0.001^*∗*^	3.530	1.642–11.965	0.022
Size of the nodules	0.301			

OR: odds ratio.

## Data Availability

The data used to support the findings of this study are included in the manuscript.
